# Diagnostic and prognostic performance to detect Alzheimer’s disease and clinical progression of a novel assay for plasma p-tau217

**DOI:** 10.1186/s13195-022-01005-8

**Published:** 2022-05-14

**Authors:** Colin Groot, Claudia Cicognola, Divya Bali, Gallen Triana-Baltzer, Jeffrey L. Dage, Michael J. Pontecorvo, Hartmuth C. Kolb, Rik Osssenkoppele, Shorena Janelidze, Oskar Hansson

**Affiliations:** 1 Clinical Memory Research Unit, Department of Clinical Sciences, Skånes universitetssjukhus, VE Minnessjukdomar, Lund University, 205 02 Malmö, Sweden; 2 Neuroscience Biomarkers, Janssen Research & Development, La Jolla, CA USA; 3 Stark Neurosciences Research Institute, Indiana University School of Medicine, Indianapolis, IN USA; 4 Avid Radiopharmaceuticals, Philadelphia, PA USA; 5 Eli Lilly and Company, Indianapolis, IN USA; 6 Alzheimer center, Amsterdam UMC location VUmc, Amsterdam, The Netherlands

**Keywords:** Alzheimer’s disease, Mild cognitive impairment, Plasma biomarkers, p-tau, Assay

## Abstract

**Background:**

Recent advances in disease-modifying treatments highlight the need for accurately identifying individuals in early Alzheimer’s disease (AD) stages and for monitoring of treatment effects. Plasma measurements of phosphorylated tau (p-tau) are a promising biomarker for AD, but different assays show varying diagnostic and prognostic accuracies. The objective of this study was to determine the clinical performance of a novel plasma p-tau217 (p-tau217) assay, p-tau217+_Janssen_, and perform a head-to-head comparison to an established assay, plasma p-tau217_Lilly_, within two independent cohorts_._

**Methods:**

The study consisted of two cohorts, cohort 1 (27 controls and 25 individuals with mild-cognitive impairment [MCI]) and cohort 2 including 147 individuals with MCI at baseline who were followed for an average of 4.92 (SD 2.09) years. Receiver operating characteristic analyses were used to assess the performance of both assays to detect amyloid-β status (+/−) in CSF, distinguish MCI from controls, and identify subjects who will convert from MCI to AD dementia. General linear and linear mixed-effects analyses were used to assess the associations between p-tau and baseline, and annual change in Mini-Mental State Examination (MMSE) scores. Spearman correlations were used to assess the associations between the two plasma measures, and Bland-Altmann plots were examined to assess the agreement between the assays.

**Results:**

Both assays showed similar performance in detecting amyloid-β status in CSF (plasma p-tau217+_Janssen_ AUC = 0.91 vs plasma p-tau217_Lilly_ AUC = 0.89), distinguishing MCI from controls (plasma p-tau217+_Janssen_ AUC = 0.91 vs plasma p-tau217_Lilly_ AUC = 0.91), and predicting future conversion from MCI to AD dementia (plasma p-tau217+_Janssen_ AUC = 0.88 vs p-tau217_Lilly_ AUC = 0.89). Both assays were similarly related to baseline (plasma p-tau217+_Janssen_ rho = −0.39 vs p-tau217_Lilly_ rho = −0.35), and annual change in MMSE scores (plasma p-tau217+_Janssen_*r* = −0.45 vs p-tau217_Lilly_*r* = −0.41). Correlations between the two plasma measures were rho = 0.69, *p* < 0.001 in cohort 1 and rho = 0.70, *p* < 0.001 in cohort 2. Bland-Altmann plots revealed good agreement between plasma p-tau217+_Janssen_ and plasma p-tau217_Lilly_ in both cohorts (cohort 1, 51/52 [98%] within 95%CI; cohort 2, 139/147 [95%] within 95%CI).

**Conclusions:**

Taken together, our results indicate good diagnostic and prognostic performance of the plasma p-tau217+_Janssen_ assay, similar to the p-tau217_Lilly_ assay.

**Supplementary Information:**

The online version contains supplementary material available at 10.1186/s13195-022-01005-8.

## Background

Alzheimer’s disease (AD) biomarkers are essential for establishing an accurate diagnosis and prognosis, and for participant selection for clinical trials [1–3]. Furthermore, recent advances in disease-modifying treatments highlight the need for accurately identifying individuals in early AD stages who are likely to benefit from particular interventions, and for monitoring treatment effects [4–6]. Non-invasive, cost-effective, and accessible plasma biomarkers for AD are promising candidates to meet that need [7].

Several blood biomarkers are currently available that are able to detect AD pathological changes or their downstream effects, but among the most promising in AD research is phosphorylated tau (p-tau). P-tau has been shown to (1) detect AD pathology, (2) discriminate AD from non-AD, and (3) accurately identify AD already in the preclinical stages of the disease [7–15]. A range of p-tau isoforms can be detected in plasma, including p-tau181, p-tau202, p-tau217, and p-tau231, which show varying dynamic ranges and diagnostic and prognostic accuracies [12–14, 16, 17]. Consistent with comparisons between p-tau217 and p-tau181 in CSF [18, 19], plasma p-tau217 is shown to perform slightly better than plasma p-tau181 in terms of detecting AD pathology and AD dementia [12, 20, 21]. However, the question remains whether different assays that are available to measure plasma p-tau217 yield comparable results. A previous examination that assessed a range of p-tau measures reported that the correlation between p-tau181 measured on the Mesoscale Scale Discovery (MSD) platform and p-tau181 measured using a single-molecule array (Simoa) was only modest (*r* = 0.66) [16]. This indicates that p-tau measurements are influenced by the platform and/or capture and detection antibodies that are used, but a direct comparison of plasma p-tau217 assays has not yet been performed.

A previous study that assessed ~ 1400 plasma p-tau217 samples (across 4 cohorts) using an immunoassay on an MSD platform developed by Lilly Research Laboratories (plasma p-tau217_Lilly_) showed that plasma p-tau217_Lilly_ discriminated between AD and other neurodegenerative diseases with effects sizes that were not significantly different from CSF p-tau217 and tau-PET [12]. A novel plasma p-tau217 measure, plasma p-tau217+_Janssen_, was recently developed by Janssen Research & Development. This measure is quantified with a Simoa assay and is enhanced by concomitant phosphorylation at aa 212 (indicated by “+”). In an initial investigation, plasma p-tau217+_Janssen_ was shown to exhibit good technical performance, discriminate accurately between AD and controls, and detect Aβ-positive and CSF p-tau-positive participants [22]. However, the plasma p-tau217+_Janssen_ assay has not yet been validated in a large clinical cohort with amyloid-β (Aβ)-positivity or progression to AD dementia as outcome measures. The primary aim of the present study was therefore to study the clinical performance of the plasma p-tau217+_Janssen_ assay and to compare it with the p-tau217_Lilly_ assay, which is one of the best performing assays to date [12, 16, 18, 23]. To this end, we assessed the diagnostic and prognostic accuracy of plasma p-tau217+_Janssen_ and plasma p-tau217_Lilly_ in a head-to-head comparison within two independent cohorts.

## Methods

The objective of this case-control, observational study is to determine the clinical performance of a novel plasma p-tau217 (p-tau217) assay, p-tau217+_Janssen_, and perform a head-to-head comparison to an established assay, plasma p-tau217_Lilly_, within two independent cohorts_._

### Participants

#### Cohort 1

A cross-sectional cohort of cognitively unimpaired individuals (controls) and individuals with mild cognitive impairment (MCI) due to AD was selected from the Swedish BioFINDER study. The inclusion criteria for controls were the absence of objectifiable cognitive symptoms and not fulfilling the criteria for MCI [24] or any dementia disorder. The inclusion criteria for individuals with MCI were based on the clinical criteria by Petersen [24]: (1) being referred to a memory clinic because of cognitive complaints, (2) CSF Aβ-positive (CSF-Aβ+) as defined by CSF Aβ_42_/Aβ_40_ ratios (see the “Plasma and CSF analyses” section), (3) age 60–80 years, (4) objective cognitive impairment, and (4) not fulfilling the criteria for any dementia disorder. The exclusion criteria for both groups were (1) significant unstable systemic illness or organ failure, (2) current significant alcohol or substance misuse, and (3) cognitive impairment that could be explained by other specific non-neurodegenerative disorders such as brain tumor or subdural hematoma. This yielded a cohort of 52 subjects, consisting of 27 cognitively unimpaired individuals and 25 with MCI.

#### Cohort 2

The second, longitudinal cohort was selected at the Memory Clinic at Skåne University Hospital in Malmö, Sweden, and included individuals who were clinically diagnosed with MCI at baseline. This cohort has previously been described in detail [25, 26]. Participants with MCI were included in the present study based on the same criteria as in cohort 1 [24] and a Mini-Mental Status Examination (MMSE) score ≥ 24. These criteria resulted in a sample of 147 participants with MCI (at baseline) who were followed up for an average of 4.8 (SD 2.1; median 4.73) years. Participants in this cohort were stratified into subgroups based on their clinical diagnosis at the last follow-up visit. A classification of MCI-AD was assigned when the participant progressed to AD dementia based on the DSM-IIIR criteria for dementia and the NINDS-ADRDA criteria for probable AD [27, 28]. MCI-AD participants were also required to be Aβ-positive according to CSF-Aβ42/40 ratios (see the “Plasma and CSF analyses” section). Participants that developed dementia, but not AD dementia, were classified as MCI-other. Participants who did not progress to dementia were classified as “stable MCI.” All of these groups were further divided into groups that were Aβ-positive and Aβ-negative (aside from the MCI-AD group, who were all Aβ-positive). This resulted in the following groups MCI-AD Aβ+ (*n* = 45), MCI-other Aβ− (*n* = 24; vascular dementia [VaD; *n* = 14], progressive supranuclear palsy [PSP; *n* = 2], normal pressure hydrocephalus [NPH; *n* = 1], dementia with Lewy bodies [DLB]; *n* = 3, AD-type dementia but Aβ-negative; *n* = 4), MCI-other Aβ+ (*n* = 9; DLB *n* = 1, semantic dementia [SD] *n* = 1, PSP *n* = 1, VaD *n* = 6), Stable MCI Aβ− (*n* = 51) and stable MCI Aβ+ (*n* = 18).

### Measures

*N* of measurements across variables and cohorts is provided in Additional file 1: Table S1.

### Plasma and CSF sampling

Plasma and CSF samples were gathered in the morning while participants were non-fasting. In order to obtain plasma, blood was collected in six K^2^-EDTA-plasma tubes and centrifuged (2000*g*, +4 °C) for 10 min. After centrifugation, the plasma was aliquoted into 1.5-ml polypropylene tubes (1-ml plasma in each tube) and stored at − 80 °C within 30–60 min of blood collection. CSF was obtained by lumbar puncture and stored at −80 °C in polypropylene tubes following the Alzheimer’s Association flow chart for lumbar puncture and CSF sample processing [29].

### Plasma and CSF analyses

**Fig. 1:**
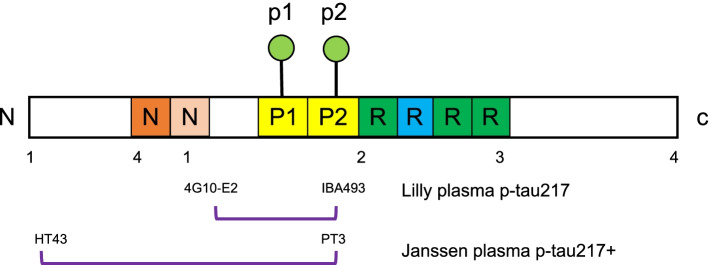
Overview of the antibodies used in the 2 plasma p-tau217 assays. Plasma p-tau217+_Janssen_ uses a single molecule array (Simoa) and the + highlights that PT3 binding requires phosphorylation at aa217 and is enhanced from additional phosphorylation at aa212. Plasma p-tau217_Lilly_ uses the Meso Scale Discovery (MSD) platform

Plasma and CSF concentrations of p-tau217 were measured using two novel single-molecule array (Simoa) assays developed by Janssen Research and Development ( [22] for plasma and [30] for CSF) and with an immunoassay on an MSD platform developed by Lilly Research Laboratories as previously described [12]. For p-tau217_Lilly_ (which was analyzed at Lund University, Sweden), biotinylated-IBA493 (anti-p-tau217) was used as a capture antibody and SULFO-TAG-4G10-E2 (anti-tau) as the detector; plasma and CSF samples were diluted 1:2 and 1:4, respectively; the assay was calibrated with a synthetic p-tau217 peptide. For plasma p-tau217+_Janssen_ (which was analyzed at Janssen, USA), PT3 (binding requires phosphorylation at aa217 and is enhanced from additional phosphorylation at aa212) was used as a capture antibody and HT43 (anti-tau) as the detector. From 250-μl plasma aliquot, 230 μl was loaded into the assay, and samples were diluted 1:2.6. CSF samples were diluted 1:8. The assay was calibrated with a synthetic p-tau212/217 peptide (Fig. 1). Analytical performance of the plasma p-tau217+_Janssen_ and plasma p-tau217_Lilly_ assays is outlined in Additional file 1: Text S1.

CSF Aβ_40_ and Aβ_42_ levels were assessed by electrochemiluminescence technology (Meso Scale Discovery [MSD], Gaithersburg, MD, USA), using the MS6000 Human Abeta 3-Plex Ultra-Sensitive Kit, following the manufacturer’s recommendations. CSF amyloid-positivity (CSF-Aβ+) was defined by a CSF Aβ_42_/Aβ _40_ ratio below 0.07. This cutoff was determined in previous work using the Youden index for optimal separation of AD dementia participants from cognitively healthy controls [25, 26].

### Amyloid-PET

Amyloid-PET using [^18^F]flutemetamol was performed in 44 out of 52 subjects of cohort 1 (Additional file 1: Table S1). Images were acquired on GE Discovery MI scanners after injection of ~185 MBq [^18^F]flutemetamol as previously described [31]. Sum images (from 90–110 min post-injection) were analyzed using the software NeuroMarQ (GE Healthcare, Cleveland, OH, USA). [^18^F]flutemetamol uptake was assessed with a previously described fully automated PET-only method that uses an adaptive template for handling differential uptake patterns in negative and positive [^18^F]flutemetamol scans [32]. [^18^F]flutemetamol images were then spatially normalized using the adaptive template method. A volume of interest (VOI) template was applied to obtain a global neocortical composite region [32] and the standardized uptake value ratio (SUVR) in this VOI was defined by normalizing for cerebellar cortex uptake. Amyloid-PET-positivity was based on a previously defined cutoff in the global VOI (>1.42 = amyloid-PET+) [31]. All subjects were concordant on CSF and amyloid-PET, except for one control which was CSF-Aβ+ but amyloid-PET−.

### Statistical analyses

All analyses and visualizations were performed using *R* software version 4.0.3. For both cohorts, values from plasma p-tau217+_Janssen_ and plasma p-tau217_Lilly_ levels were log-transformed in parametric analyses. To allow for easier visual comparison between the two assays, *z*-scored values are displayed in some of the figures (see figure legends), but the values were not *z*-transformed when entered into the statistical analyses.

#### Cohort 1

General linear models and least significant differences (LSD) post hoc tests (adjusted for age and sex) and receiver operating characteristic (ROC) analysis (R package “pROC”) were used to assess the differences in plasma p-tau217 between (1) CSF-Aβ+ and CSF-Aβ− subjects, (2) between amyloid-PET+ and amyloid-PET− subjects, and (3) between individuals clinically diagnosed as cognitively unimpaired vs diagnosed as MCI. To test whether two area under the curve (AUC) statistics were significantly different, we used the DeLong method. Spearman correlations were used to assess the association between plasma measures, and Bland-Altmann plots were assessed in order to examine the agreement between plasma measures.

#### Cohort 2

Plasma p-tau217 levels were compared between CSF-Aβ+ and CSF-Aβ− subjects and between diagnostic groups that were stratified according to diagnosis at follow-up as well as for amyloid status (MCI-AD [Aβ+], MCI-other Aβ−, MCI-other Aβ+, stable MCI Aβ−, and stable MCI Aβ+). For these comparisons, we again used general linear models (adjusted for age, sex, and [for comparing diagnostic groups] total follow-up time) and ROC analyses (DeLong method). The Youden index with bootstrapping (100 repeats) was used to determine AUC, accuracy, sensitivity, and specificity with 95% confidence interval (CI) at optimal cut-points for both assays. Additionally, binary logistic regression analyses were used to assess the effects of plasma p-tau217 on Aβ-status and progression to AD dementia (yes/no) while adjusting for age, sex, and APOEϵ4 carriership. Spearman correlations and Bland-Altmann plots were assessed in order to examine the agreement between plasma measures.

##### Longitudinal effects

Cohort 2 contained longitudinal measurements of plasma samples (average follow-up 4.92 [2.09] years), which were used to examine the differences in change over time on the two plasma p-tau217 assays (i.e., the effect of time on change in p-tau217). Change over time in plasma p-tau217 was calculated by subtracting the baseline value from the last follow-up value, giving Δp-tau217. Furthermore, general linear models with post hoc LSD tests were used to compare Δp-tau217 between the diagnostic groups, with adjustment for age, sex, and total follow-up time. Finally, longitudinal measures of MMSE were used in general linear models to assess the effect of baseline p-tau217 on ΔMMSE between baseline and follow-up, adjusted for age and sex.

## Results

In cohort 1, plasma p-tau217+_Janssen_ and plasma p-tau217_Lilly_ levels were higher in the MCI group than in the controls. APOEϵ4 carriers, CSF-Aβ+ subjects, and amyloid-PET+ subjects in cohort 1 were all more prevalent in the MCI group than in the control group. As expected, in cohort 2, there were also differences between the diagnostic groups in age, APOEϵ4 carriership, MMSE, and plasma p-tau217+_Janssen_ and plasma p-tau217_Lilly_ levels. Unfortunately, the follow-up time in cohort 2 was not consistent across the groups (Table 1 and Additional file 1: Table S2).

**Table 1:** Baseline demographic and clinical characteristics of the samples

**Cohort 1**	**Overall**	**Control**	**MCI-AD (Aβ+)**	***p***			
*n*	52	27	25				
Age	72.13 (5.54)	72.63 (5.38)	71.60 (5.77)	0.509			
Sex, female	25 (48.1)	10 (37.0)	15 (60.0)	0.168			
APOEϵ4 positive	25 (48.1)	6 (22.2)	19 (76.0)	< 0.001			
Education	11.40 (2.97)	11.63 (2.44)	11.16 (3.48)	0.573			
Plasma p-tau217+_Janssen_, pg/ml	0.05 (0.04)	0.03 (0.01)	0.08 (0.05)	< 0.001			
Plasma p-tau217_Lilly_, pg/ml	0.43 (0.21)	0.31 (0.05)	0.56 (0.23)	< 0.001			
**Cohort 2**	**Overall**	**MCI-AD (Aβ+)**	**MCI-other Aβ−**	**MCI-other Aβ+**	**Stable MCI Aβ−**	**Stable MCI Aβ+**	***p*** **across the groups**
*n*	147	45	24	9	51	18	
Age	72.06 (7.71)	75.89 (6.97)	73.12 (7.60)	71.67 (5.36)	68.84 (7.70)	70.39 (6.70)	< 0.001
Sex, female	86 (58.5)	34 (75.6)	11 (45.8)	3 (33.3)	29 (56.9)	9 (50.0)	0.042
APOEϵ4 positive	82 (55.8)	36 (80.0)	11 (45.8)	7 (77.8)	14 (27.5)	14 (77.8)	< 0.001
MMSE	27.37 (1.78)	26.09 (1.65)	27.00 (1.82)	27.67 (2.06)	28.45 (1.05)	27.83 (1.47)	< 0.001
Total follow-up, years	4.92 (2.09)	3.66 (1.63)	3.90 (1.97)	4.49 (2.00)	6.34 (1.70)	5.64 (1.65)	< 0.001
Plasma p-tau217+_Janssen_, pg/ml	0.08 (0.07)	0.13 (0.08)	0.05 (0.04)	0.09 (0.07)	0.04 (0.03)	0.07 (0.04)	< 0.001
Plasma p-tau217_Lilly_, pg/ml	0.30 (0.18)	0.46 (0.18)	0.23 (0.11)	0.30 (0.15)	0.20 (0.12)	0.31 (0.11)	< 0.001
CSF p-tau217+_Janssen_, pg/ml	11.66 (16.29)	25.29 (23.38)	4.72 (3.75)	6.61 (3.52)	3.58 (2.38)	12.27 (7.22)	< 0.001
CSF p-tau217_Lilly_, pg/ml	17.83 (22.92)	38.28 (30.62)	8.03 (6.96)	10.02 (4.49)	5.09 (3.26)	19.80 (14.60)	< 0.001

Values displayed are mean (SD) for continuous variables and *n* (%) for categorical variables. Pairwise differences between the groups are displayed in Additional file 1: Table S2

*Aβ* amyloid-β, *MMSE* Mini-Mental State Examination, *MCI* mild cognitive impairment, *p-tau* phosphorylated tau

### Plasma p-tau217 between Aβ+ and Aβ− subjects

#### Cohort 1

**Fig. 2:**
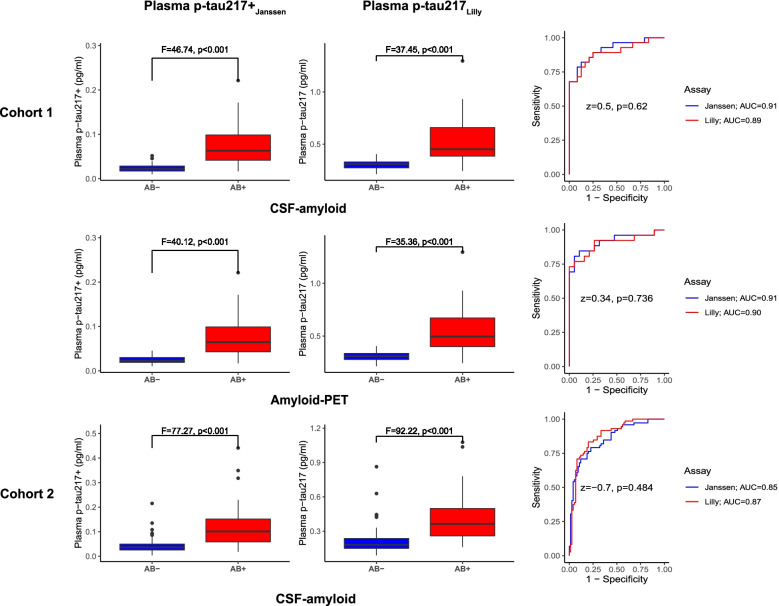
Differences in plasma p-tau217 according to amyloid status. CSF-Aβ+ was determined by CSF Aβ42/Aβ40 ratio < 0.07, and amyloid-PET+ was determined by >1.42 SUVR. AUC, area under the curve

Baseline plasma p-tau217 levels from both assays were compared between CSF-Aβ+ and CSF-Aβ− participants as well as between amyloid-PET+ and amyloid-PET− participants. Both plasma p-tau217+_Janssen_ and plasma p-tau217_Lilly_ were higher in CSF-Aβ+ subjects with a mean fold increase (mean CSF-Aβ+ minus mean CSF-Aβ− divided by mean CSF-Aβ−) of 2.1 (Cohen’s *d* [95%CI] = 1.44 [0.81–2.10]) and 0.8 (*d* = 1.35 [0.73–1.97]) for plasma p-tau217+_Janssen_ and plasma p-tau217_Lilly_, respectively. The AUC for plasma p-tau217+_Janssen_ to distinguish CSF-Aβ+ from CSF-Aβ− was AUC (95%CI) = 0.91 (0.84–0.99), and the corresponding AUC for plasma p-tau217_Lilly_ was 0.89 (0.80–0.98), and these AUCs were not significantly different (*z* = 0.50, *p* = 0.620). In cohort 1, baseline plasma p-tau217 levels from both assays were also higher in amyloid-PET+ subjects (plasma p-tau217+_Janssen_, 2.1-fold increase, Cohen’s *d* [95%CI] = 1.42 [0.74–2.10], between amyloid-Aβ− and amyloid-Aβ+; plasma p-tau217_Lilly_, 0.8-fold increase, *d* = 1.36 [0.68–2.03]). The AUC for plasma p-tau217+_Janssen_ to distinguish amyloid-PET+ from amyloid-PET− was AUC (95%CI) = 0.91(0.83–1.00), and the corresponding AUC for plasma p-tau217_Lilly_ was 0.90 (0.81–1.00), and these were not significantly different (*z* = 0.34, *p* = 0.736; Fig. 2).

#### Cohort 2

Baseline plasma p-tau217 levels from both assays were compared between CSF-Aβ+ and CSF-Aβ− participants which revealed that both plasma p-tau217+_Janssen_ and plasma p-tau217_Lilly_ were higher in CSF-Aβ+ subjects (plasma p-tau217+_Janssen_, 2.6-fold increase, Cohen’s *d* [95%CI] = 1.21 [0.86–1.56], between CSF-Aβ− and CSF-Aβ+; plasma p-tau217+_Lilly_, 1.9-fold increase, *d* = 1.28 [0.92–1.63]). The AUC for plasma p-tau217+_Janssen_ to distinguish CSF-Aβ+ from CSF-Aβ− was AUC (95%CI) = 0.85(0.79–0.91), and the corresponding AUC for plasma p-tau217_Lilly_ was 0.87 (0.82–0.93), and these were not significantly different (*z* = 0.70, *p* = 0.484; Fig. 2).

### Plasma p-tau217 between the diagnostic groups

#### Cohort 1

**Fig. 3:**
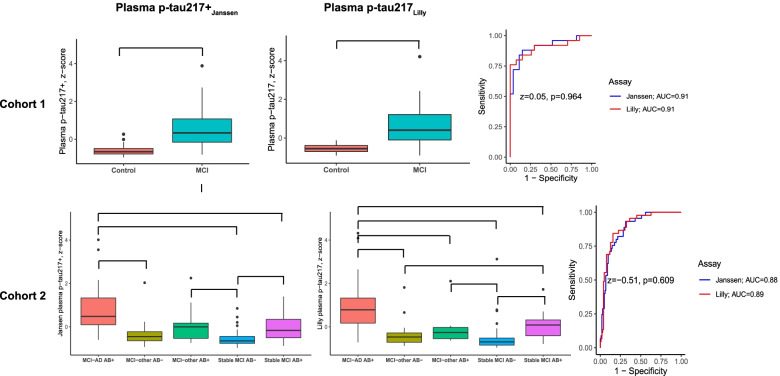
Differences in plasma p-tau217 according to diagnostic groups. The groups in cohort 2 were stratified according to amyloid status (Aβ+ = CSF Aβ42/Aβ40 ratio < 0.07) and clinical diagnosis at follow-up (e.g., MCI-AD Aβ+ has AD dementia at follow-up). Brackets indicate significant differences between the groups, determined by general linear models with post hoc LSD tests, adjusted for age, sex, and [in cohort 2] total follow-up time. The *y*-axes represent the *z*-scored plasma p-tau217 levels in order to facilitate an easier visual comparison between the two assays. AUC, area under the curve

Comparing plasma p-tau217 between controls and MCI subjects, we found that MCI subjects had significantly higher plasma p-tau217+_Janssen_ (2.0 fold increase [Cohen’s *d* [95%CI] = 1.55 [0.91–2.19]) and plasma p-tau217_Lilly_ (0.4 fold increase, *d* = 1.51 [0.88–2.14]) levels than controls. The performance of plasma p-tau217+_Janssen_ and plasma p-tau217_Lilly_ to distinguish MCI from controls was similar (AUC [95%CI] plasma p-tau217+_Janssen_ = 0.91 [0.82–0.99], plasma p-tau217_Lilly_ = 0.91 [0.82–1.00], *z* = 0.05, *p* = 0.964; Fig. 3).

#### Cohort 2

Comparing plasma p-tau217 between the diagnostic groups who were stratified according to amyloid status and clinical diagnosis at follow-up, we found that MCI-AD had significantly higher plasma p-tau217+_Janssen_ levels than all other groups except MCI-other Aβ+ (fold increase compared to MCI-other Aβ− 1.5, Cohen’s *d* [95%CI] = 1.17 [0.63–1.71]; stable MCI Aβ− 2.5, *d* [95%CI] = 1.64 [1.17–2.10]; stable MCI Aβ+ 0.8, *d* = 0.83 [0.26–1.41]). Plasma p-tau217_Lilly_ was higher in MCI-AD compared to all other groups (fold increase compared to MCI-other Aβ− 1.0, Cohen’s *d* [95%CI] = 1.45 [0.89–2.01]; MCI-other Aβ+ 0.6, *d* = 1.38 [0.89–1.87]; stable MCI Aβ− 1.3, *d* = 1.71 [1.24–2.19]; stable MCI Aβ+ 0.5, *d* = 0.94 [0.36–1.52]). The AUCs for the two assays to detect future progression from MCI to AD dementia (i.e., distinguish the MCI-AD group from the other groups) were found to not be significantly different (AUC [95%CI] plasma p-tau217+_Janssen_ = 0.88 [0.82–0.93], plasma p-tau217_Lilly_ = 0.89 [0.83–0.95], *z* = − 0.51, *p* = 0.609; Fig. 3).

In cohort 2, we also assessed whether the similarities between AUC for plasma p-tau217+_Janssen_ and plasma p-tau217_Lilly_ were impacted when also considering age, sex, and APOEϵ4 carriership when detecting CSF-Aβ+ and predicting future conversion to AD dementia. We found that, as expected, AUC values increased when also considering these factors, and they became even more similar between the two assays (Additional file 1: Table S3). Additionally, we determined the optimal cut-points for plasma p-tau217+_Janssen_ and plasma p-tau217_Lilly_ to detect CSF-Aβ+ and future conversion to AD dementia and examined the sensitivity and specificity of the two assays at these optimal cut-points. Sensitivity and specificity to detect CSF-Aβ+ and conversion to AD dementia were 0.75/0.85 and 0.84/0.81, respectively, for plasma p-tau217+_Janssen_, while the corresponding sensitivity and specificity for plasma p-tau217_Lilly_ were 0.81/0.85 and 0.85/0.84, respectively (Additional file 1: Table S4).

### Longitudinal effects

#### Cohort 2

We found that plasma p-tau217+_Janssen_ as well as plasma p-tau217_Lilly_ increased with time in the whole sample, and change over time was not different between the assays (plasma p-tau217+_Janssen_*r* = 0.35, *p* < 0.001; plasma p-tau217_Lilly_*r* = 0.14, *p* < 0.001; *z* = 1.54, *p* = 0.124). We also found no differences in the change over time between the assays when assessing the MCI-AD group separately (plasma p-tau217+_Janssen_*r* = 0.50, *p* < 0.001; plasma p-tau217_Lilly_*r* = 0.53, *p* < 0.001; *z* = 0.14, *p* = 0.889) and in participants that did not convert to AD dementia (i.e., all groups combined, except MCI-AD; plasma p-tau217+_Janssen_*r* = 0.34, *p* < 0.001, plasma p-tau217_Lilly_*r* = 0.09, *p* = 0.031; *z* for difference = 1.58, *p* = 0.114).

**Fig. 4:**
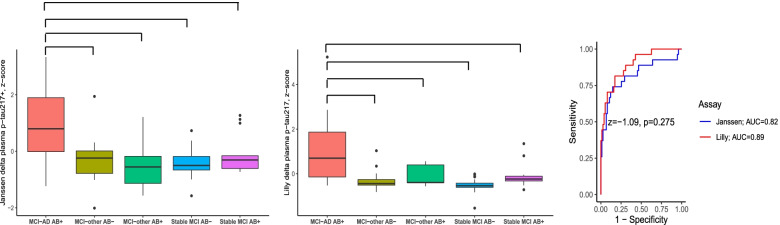
Differences in Δ plasma p-tau217 across the diagnostic groups, cohort 2 only. The groups were stratified according to amyloid status determined by the Aβ42/Aβ40 ratio and by clinical diagnosis at follow-up (e.g., MCI-AD Aβ+ has AD dementia at follow-up). Brackets indicate a significant difference between the groups, determined by general linear models with post hoc LSD tests, adjusted for age, sex, and total follow-up time. Plasma p-tau217 levels were *z*-scored to facilitate comparison between plasma p-tau217+_Janssen_ and plasma p-tau217_Lilly_ assays. AUC, area under the curve

We also assessed the differences in change over time (Δ/year) in plasma p-tau217 across the diagnostic groups. General linear models with LSD post hoc tests revealed that the MCI-AD group had a significantly higher annual increase compared to all other groups, and this was true both for plasma p-tau217+_Janssen_ (fold increase compared to MCI-other Aβ− 2.2, Cohen’s *d* [95%CI] = 1.07 [0.38–1.77]; MCI-other Aβ+ 3.6, *d* = 1.04 [0.43 1.65]; stable MCI Aβ− 3.3, *d* = 1.60 [1.04–2.16]; stable MCI Aβ+ 1.4, *d* = 0.91 [0.20–1.62]) and plasma p-tau217_Lilly_ (fold increase compared to MCI-other Aβ− 5.2, Cohen’s *d* [95%CI] = 1.21 [0.51–1.91]; MCI-other Aβ+ 2.4, *d* = 1.12 [0.51–1.74]; stable MCI Aβ− 43.2, *d* = 1.84 [1.25–2.42]; stable MCI Aβ+ 2.3, *d* = 0.96 [0.22–1.70]; Fig. 4). ROC analyses also revealed that annual change in plasma p-tau217+_Janssen_ and plasma p-tau217_Lilly_ were similarly able to distinguish converters to AD dementia from those not converting to AD dementia (Δ plasma p-tau217+_Janssen_ AUC = 0.82 [0.71–0.93]; Δ plasma p-tau217_Lilly_ AUC = 0.89 [0.82–0.96]; *z* for difference = − 1.09, *p* = 0.275; Fig. 4), and the difference between AUC statistics was smaller when also considering age, sex, and APOEϵ4 carriership (Δ plasma p-tau217+_Janssen_ AUC = 0.94 [0.88–0.99]; Δ plasma p-tau217_Lilly_ AUC = 0.95 [0.91–0.99]; *z* for difference = − 1.09, *p* = 0.275).

### Associations between the plasma p-tau217 measures

**Fig. 5:**
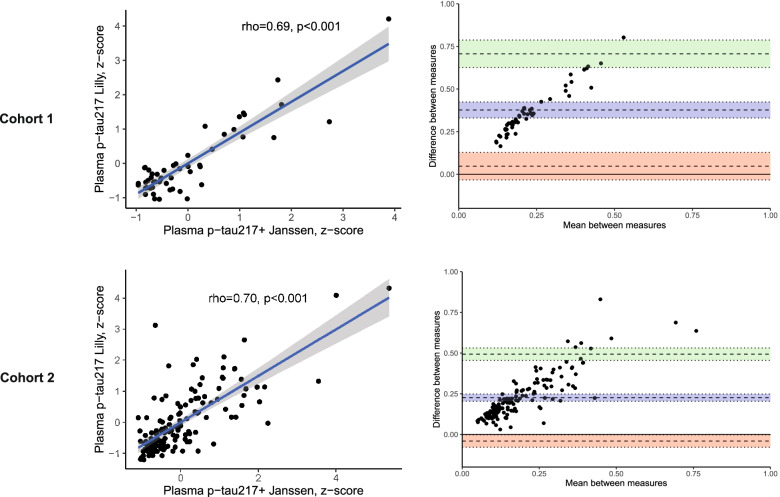
Spearman correlation analyses and Bland-Altmann plots assessing the associations and agreement between plasma p-tau217 assays. The figure displays the plasma vs plasma Spearman’s (rho) correlations between plasma p-tau217+_Janssen_ and plasma p-tau217_Lilly_, as well as Bland-Altmann plots that visualize the agreement between the plasma measures. In the Bland-Altmann plots, the blue line represents the mean difference, and the dotted green and red lines represent 95% confidence intervals (CI). Plasma p-tau217 levels in the scatter plots were *z*-scored in order to facilitate comparison between plasma p-tau217+_Janssen_ and plasma p-tau217_Lilly_ values

In cohort 1, the correlation between the two plasma measures was rho = 0.69, *p* < 0.001. In cohort 2, the correlation between the two plasma measures was rho = 0.70, *p* < 0.001 (Fig. 5), and the correlation between CSF measures (CSF p-tau217+_Janssen_ and CSF p-tau217_Lilly_) was rho = 0.98, *p* < 0.001. We also assessed the agreement between the two plasma measures using Bland-Altmann plots, which revealed good agreement between plasma p-tau217+_Janssen_ and plasma p-tau217_Lilly_ in both cohorts (cohort 1, 51/52 [98%] within 95%CI; cohort 2, 139/147 [95%] within 95%CI), with agreement decreasing at higher plasma p-tau_217_ levels (Fig. 5). In cohort 2, the correlation between CSF and plasma measurements of p-tau217 as assessed by the p-tau217+_Janssen_ assay was rho = 0.62, *p* < 0.001, and the corresponding correlation for measurements with the p-tau217_Lilly_ assay was rho = 0.68, *p* < 0.001. Fisher’s *z*-test showed that these correlation coefficients did not statistically differ (*z* = 1.07, *p* = 0.285).

### Associations with MMSE

#### Replication cohort

Both baseline plasma p-tau217+_Janssen_ and plasma p-tau217_Lilly_ were correlated to baseline MMSE (plasma p-tau217+_Janssen_ rho = −0.39, *p* < 0.001; plasma p-tau217_Lilly_ rho = −0.35, *p* < 0.001) and to annual change in MMSE (plasma p-tau217+_Janssen_*r* = −0.45, *p* < 0.001; plasma p-tau217_Lilly_*r* = −0.41, *p* < 0.001). There were no differences in terms of associations with baseline (*z* for difference = 0.44, *p* = 0.657) or longitudinal change in MMSE (*z* for difference = 0.43, *p* = 0.667) between the two assays (Additional file 1: Fig. S1).

### Sensitivity analyses

There were a few subjects with baseline plasma p-tau217 levels that would be considered outliers according to the threshold (mean ± 3SD, Additional file 1: Fig. S2). In order to assess whether these cases affected our results, we removed 1 MCI case from cohort 1 based on the plasma p-tau217_Lilly_ value being an outlier and 6 outlier cases (plasma p-tau217+_Janssen_*n* = 2, plasma p-tau217_Lilly_*n* = 3, and plasma p-tau217+_Janssen_ and plasma p-tau217+_Lilly_*n* = 1; Additional file 1: Fig. S2) from cohort 2 and reran all analyses. There were no differences in the results for cohort 1. In the analyses in cohort 2 without the outliers, baseline plasma p-tau217+_Janssen_ was significantly higher in MCI-AD than in MCI-other Aβ+, while it was not in the initial analysis. Additionally, for cohort 2 only, we also reran all analyses after removing stable MCI Aβ− and stable MCI Aβ+ participants who had less than 5 years of follow-up. In these analyses, Δ plasma p-tau217+_Janssen_ was no longer different between MCI-AD and MCI-other Aβ+.

## Discussion

In the present study, we used two independent cohorts to assess the diagnostic and prognostic performance of a novel plasma p-tau217 assay, p-tau217+_Janssen_ (Simoa), by performing a head-to-head comparison against plasma p-tau217_Lilly_ (MSD platform). We observed that both assays performed similarly in terms of detecting amyloid status, distinguishing MCI subjects from controls, and detecting MCI subjects who will go on to develop AD dementia. Furthermore, we found that additionally considering age, sex, and APOEϵ4 carriership not only increased the effect sizes but also resulted in even more similar effect sizes between the assays. This similarity in the performance of the two assays also extended to longitudinal change in plasma p-tau217 and to the associations of plasma p-tau217 with baseline and longitudinal MMSE measurements. We did observe that plasma p-tau217+_Janssen_ levels were not different between MCI subjects that went on to develop AD dementia and MCI subjects who developed dementia but not AD dementia and were Aβ-positive. On the other hand, we observed that plasma p-tau217+_Janssen_ had a higher fold change between CSF-Aβ+ and CSF-Aβ− individuals, suggesting better discriminating effects than for plasma p-tau217_Lilly_. However, Cohen’s *d* effect sizes (that take into account variance) for the differences between Aβ− and Aβ+ subjects and across the diagnostic groups were similar between the assays, and the AUCs to detect CSF-Aβ+ and predict conversion to AD dementia were not different. Plus, all other results also pointed to a similar diagnostic and prognostic performance for the two assays. We therefore conclude that, based on our results, the novel plasma p-tau217+_Janssen_ assay could be considered equal to plasma p-tau217_Lilly_ in terms of diagnostic and prognostic performance.

### Previous literature

Our results from cohort 1 are in line with a previous examination using plasma p-tau217+_Janssen_, which revealed good performance to distinguish individuals with clinically diagnosed AD from controls, and performance to detect CSF Aβ+ subjects [22]. We add to these findings by also showing that plasma p-tau217+_Janssen_ predicts cognitive decline and future conversion from MCI to AD dementia. A previous study that performed a head-to-head comparison of plasma p-tau assays and platforms revealed differences in fold change; correlations with cognition, amyloid, and tau PET; and discriminative accuracies when comparing two plasma p-tau181 measures: one using Simoa (Quanterix Corporation) and another using the MSD platform (Lilly research laboratories) [16]. These findings indicate that assays and platforms may have an impact on the diagnostic and prognostic performance of plasma p-tau measurements [16]. In the present study, even though the plasma p-tau217+_Janssen_ and plasma p-tau217_Lilly_ assays (1) use different platforms (Simoa vs MSD), (2) use calibrators of different molecular weights, (3) use different detection antibodies with epitopes at different sites on tau (Fig. 1), (4) detect plasma p-tau217 at different magnitudes (Table 1), (5) plasma p-tau217+_Janssen_ also detects concomitant phosphorylation at aa 212, and (6) the fold-change between Aβ+/Aβ− and converters to AD dementia/non-converters were different, the diagnostic and prognostic effects of plasma p-tau217+_Janssen_ and plasma p-tau217_Lilly_ measures were found to be similar.

### Strengths and limitations

Strengths of the current study include the assessment of two assays for plasma p-tau217 within the same subjects and across two independent cohorts, the implementation of both cross-sectional and longitudinal measurement of plasma p-tau217, and a considerable follow-up time (mean 4.92, SD 2.09) that is needed to accurately establish the prognostic value of a biomarker to detect incipient AD dementia. Limitations include sample sizes (*N* = 52 and *N* = 147), assessment of longitudinal change based on only two measurements, and group differences in follow-up time in cohort 2. Also, we limited our analyses to controls and MCI participants and did not assess the effects of the two assays in the dementia stage of AD. Lastly, although the two assays had comparable clinical performance, intra-assay coefficients of variation (CV) for plasma ptau217+_Janssen_ (20.8%) were higher than for plasma ptau217_Lilly_ (5.3%; Additional file 1: Text S1). Of note, high CVs in the plasma ptau217+_Janssen_ assay were more frequent for samples with low p-tau217 levels. The average CV for samples with plasma ptau217+_Janssen_ concentrations above 0.1 pg/ml was 13.5%. Currently, neither assay is yet clinic-grade and needs to be further optimized in the future. The need for further optimization of these plasma markers for p-tau217 is also highlighted by the relatively low correlation between plasma p-tau217 markers (rho = 0.69 [cohort 1] and 0.70 [cohort 2]), compared to the correlation between CSF p-tau217 markers (rho = 0.98 [cohort 2]). This lower correlation for plasma measures could be due to matrix effects when sample components negatively affect assay performance. Matrix effects could be much more pronounced in the blood than in CSF because blood is richer in proteins, and is a compositionally complex biological fluid. Future optimization would rely on examinations focussed on possible differences in analytical performance between the two assays, assessing the effects of matrix interference on the agreement between assays by examining purified samples at larger concentrations, delving deeper into the possible differences in detecting single (plasma p-tau217_Lilly_) vs multiple (plasma p-tau217+_Janssen_) phosphorylated epitopes, evaluating potential sensitivity differences between the assays, examining the optimal volume of plasma needed for the p-tau measurements, and examining why plasma p-tau217+_Janssen_ shows higher fold changes between Aβ−/Aβ+ and between those progressing from MCI to AD dementia and those who do not. These assessments were beyond the scope of this work.

## Conclusions

Both plasma p-tau217+_Janssen_ and plasma p-tau217_Lilly_ assays for plasma p-tau217 could serve to detect AD pathology, distinguish controls from MCI subjects, and predict future conversion from MCI to AD dementia. In extension, our findings suggest that treatment effect monitoring, as was recently published for the plasma p-tau217_Lilly_ assay in the TRAILBLAZER-ALZ (Donanemab) trial [5, 33], could also be achieved with the novel plasma p-tau217+_Janssen_ assay.

## Supplementary Information

**Additional file 1: Table S1.** Data availability across measures for the two cohorts. Aβ-amyloid-β, MMSE-Mini Mental State Examination, MCI-mild-cognitive impairment, p-tau- phosphorylated tau. **Table S2.** Pairwise comparisons between groups. Cohort 1: values depicted are p-values and p<0.05 signifies a significant difference. Cohort 2: depicted p-values are false discovery rate corrected and p<0.05 signifies a significant difference. Differences in continuous variables were assessed using independent samples T-tests and differences in categorical variables with Fisher’s exact tests. Red shaded cells indicate that the group on the x-axis was higher than the one on the y-axis and blue shading means the opposite. **Table S3.** Predictive effects of plasma p-tau217 when also considering age, sex and APOEϵ4 carriership. Effects were obtained by assessing the AUC of binary logistic regression models using ROC analyses. **Table S4.** Sensitivity and specificity. AUC, accuracy and sensitivity and specificity values with 95%CI were determined using the Youden index with bootstrapping (100 repeats). **Figure S1.** Associations with cognition. The top panel shows Spearman correlation analyses between baseline plasma p-tau217 of both assays and baseline MMSE. The bottom panel displays the associations between baseline plasma p-tau217 from both assays and annual change in MMSE, adjusted for age and sex. The z-statistics indicates results from a Fisher’s exact test assessing the difference between the correlation coefficients. **Figure S2.** Outliers removed for sensitivity analyses. Outlier was determined by mean+/-3SD within diagnostic groups and denoted with an X. **Text S1.** Analytic performance of plasma p-tau217+_Janssen_ and plasma p-tau217_Lilly_ in cohort 2.

## Data Availability

Anonymized data will be shared by request from a qualified academic investigator for the sole purpose of replicating procedures and results presented in the article and providing that the data transfer is in agreement with EU legislation on the general data protection regulation and decisions by the Ethical Review Board of Sweden and Region Skåne, which should be regulated in a material transfer agreement.
