# Correction: Diagnostic and prognostic performance to detect Alzheimer’s disease and clinical progression of a novel assay for plasma p-tau217

**DOI:** 10.1186/s13195-022-01023-6

**Published:** 2022-06-13

**Authors:** Colin Groot, Claudia Cicognola, Divya Bali, Gallen Triana-Baltzer, Jeffrey L. Dage, Michael J. Pontecorvo, Hartmuth C. Kolb, Rik Ossenkoppele, Shorena Janelidze, Oskar Hansson

**Affiliations:** 1 Clinical Memory Research Unit, Department of Clinical Sciences, Skånes Universitetssjukhus, VE Minnessjukdomar, Lund University, 205 02 Malmö, Sweden; 2 Neuroscience Biomarkers, Janssen Research & Development, La Jolla, CA USA; 3 Stark Neurosciences Research Institute, Indiana University School of Medicine, Indianapolis, IN USA; 4 Avid Radiopharmaceuticals, Philadelphia, PA USA; 5 Eli Lilly and Company, Indianapolis, IN USA; 6 Alzheimer Center, Amsterdam UMC location VUmc, Amsterdam, The Netherlands

**Correction: Alzheimers Res Ther 14, 67 (2022)**

**https://doi.org/10.1186/s13195-022-01005-8**

Following the publication of the original article [1] the authors reported that spelling of Dr. Rik Ossenkoppele’s last name was accidentally misspelled as “Osssenkoppele (with three s's)” rather than “Ossenkoppele”.

The original article [1] has been updated.
