# Supplementary material for: Diagnostic and prognostic performance to detect Alzheimer’s disease and clinical progression of a novel assay for plasma p-tau217
Source: Alzheimers Res Ther. 2022 May 14;14:67. doi: 10.1186/s13195-022-01005-8 (PMC9107269; doi:10.1186/s13195-022-01005-8)
Supplement: Supplementary file 1 — Additional file 1: Table S1. Data availability across measures for the two cohorts. Aβ-amyloid-β, MMSE-Mini Mental State Examination, MCI-mild-cognitive impairment, p-tau- phosphorylated tau. Table S2. Pairwise comparisons between groups. Cohort 1: values depicted are p-values and p<0.05 signifies a significant difference. Cohort 2: depicted p-values are false discovery rate corrected and p<0.05 signifies a significant difference. Differences in continuous variables were assessed using independent samples T-tests and differences in categorical variables with Fisher’s exact tests. Red shaded cells indicate that the group on the x-axis was higher than the one on the y-axis and blue shading means the opposite. Table S3. Predictive effects of plasma p-tau217 when also considering age, sex and APOEϵ4 carriership. Effects were obtained by assessing the AUC of binary logistic regression models using ROC analyses. Table S4. Sensitivity and specificity. AUC, accuracy and sensitivity and specificity values with 95%CI were determined using the Youden index with bootstrapping (100 repeats). Figure S1. Associations with cognition. The top panel shows Spearman correlation analyses between baseline plasma p-tau217 of both assays and baseline MMSE. The bottom panel displays the associations between baseline plasma p-tau217 from both assays and annual change in MMSE, adjusted for age and sex. The z-statistics indicates results from a Fisher’s exact test assessing the difference between the correlation coefficients. Figure S2. Outliers removed for sensitivity analyses. Outlier was determined by mean+/-3SD within diagnostic groups and denoted with an X. Text S1. Analytic performance of plasma p-tau217+Janssen and plasma p-tau217Lilly in cohort 2. [file 13195_2022_1005_MOESM1_ESM.docx]

# Supplemental Material

|  | Cohort 1 | Cohort 2 |
| --- | --- | --- |
| Measure | **N** | **N** |
| Baseline CSF Aβ42/40 | 52 | 145 |
| Baseline amyloid-PET | 44 | - |
| Baseline MMSE | - | 147 |
| Longitudinal MMSE | - | 145 |
| Baseline plasma p-tau217+_Janssen_ | 52 | 147 |
| Baseline plasma p-tau217_Lilly_ | 52 | 147 |
| Longitudinal plasma p-tau217+_Janssen_ | - | 103 |
| Longitudinal plasma p-tau217_Lilly_ | - | 103 |
| Baseline CSF p-tau217+_Janssen_ | - | 147 |
| Baseline CSF p-tau217_Lilly_ | - | 147 |

**Supplemental Table 1. Data availability across measures for the two cohorts**

Aβ-amyloid-β, MMSE-Mini Mental State Examination, MCI-mild-cognitive impairment, p-tau- phosphorylated tau

| **Cohort 1** |  |  |  |  |  |
| --- | --- | --- | --- | --- | --- |
|  |  | **Control** |  |  |  |
| **Age** | **MCI** | 0.510 |  |  |  |
| **Sex, female** | **MCI** | 0.164 |  |  |  |
| **APOEϵ4 positive** | **MCI** | <0.001 |  |  |  |
| **Education, years** | **MCI** | 0.510 |  |  |  |
| **Plasma p-tau217+_Janssen_** | **MCI** | <0.001 |  |  |  |
| **Plasma p-tau217_Lilly_** | **MCI** | <0.001 |  |  |  |
|  |  |  |  |  |  |
| **Cohort 2** |  |  |  |  |  |
|  |  | **MCI-AD (Aβ+)** | **MCI-other Aβ-** | **MCI-other Aβ+** | **Stable MCI Aβ-** |
| **Age** | **MCI-other Aβ-** | 0.265 | - | - | - |
|  | **MCI-other Aβ+** | 0.265 | 0.666 | - | - |
|  | **Stable MCI Aβ-** | <0.001 | 0.060 | 0.403 | - |
|  | **Stable MCI Aβ+** | 0.036 | 0.378 | 0.666 | 0.546 |
| **Sex, female** | **MCI-other Aβ-** | 0.104 | - | - | - |
|  | **MCI-other Aβ+** | 0.104 | 0.871 | - | - |
|  | **Stable MCI Aβ-** | 0.211 | 0.765 | 0.562 | - |
|  | **Stable MCI Aβ+** | 0.211 | 1.000 | 0.871 | 0.871 |
| **APOEϵ4 positive** | **MCI-other Aβ-** | 0.016 | - | - | - |
|  | **MCI-other Aβ+** | 1.000 | 0.191 | - | - |
|  | **Stable MCI Aβ-** | <0.001 | 0.191 | 0.016 | - |
|  | **Stable MCI Aβ+** | 1.000 | 0.115 | 1.000 | 0.001 |
| **MMSE** | **MCI-other Aβ-** | 0.036 | - | - | - |
|  | **MCI-other Aβ+** | 0.012 | 0.287 | - | - |
|  | **Stable MCI Aβ-** | <0.001 | <0.001 | 0.189 | - |
|  | **Stable MCI Aβ+** | <0.001 | 0.129 | 0.786 | 0.189 |
| **Total follow-up, years** | **MCI-other Aβ-** | 0.578 | - | - | - |
|  | **MCI-other Aβ+** | 0.241 | 0.434 | - | - |
|  | **Stable MCI Aβ-** | <0.001 | <0.001 | 0.008 | - |
|  | **Stable MCI Aβ+** | <0.001 | 0.004 | 0.179 | 0.208 |
| **Plasma p-tau217+_Janssen_** | **MCI-other Aβ-** | <0.001 | - | - | - |
|  | **MCI-other Aβ+** | 0.036 | 0.160 | - | - |
|  | **Stable MCI Aβ-** | <0.001 | 0.336 | 0.036 | - |
|  | **Stable MCI Aβ+** | <0.001 | 0.279 | 0.552 | 0.036 |
| **Plasma p-tau217_Lilly_** | **MCI-other Aβ-** | <0.001 | - | - | - |
|  | **MCI-other Aβ+** | 0.004 | 0.276 | - | - |
|  | **Stable MCI Aβ-** | <0.001 | 0.501 | 0.112 | - |
|  | **Stable MCI Aβ+** | <0.001 | 0.118 | 0.874 | 0.017 |
| **CSF p-tau217+_Janssen_** | **MCI-other Aβ-** | <0.001 | - | - | - |
|  | **MCI-other Aβ+** | 0.001 | 0.734 | - | - |
|  | **Stable MCI Aβ-** | <0.001 | 0.734 | 0.668 | - |
|  | **Stable MCI Aβ+** | 0.002 | 0.123 | 0.435 | 0.040 |
| **CSF p-tau217_Lilly_** | **MCI-other Aβ-** | <0.001 | - | - | - |
|  | **MCI-other Aβ+** | <0.001 | 0.779 | - | - |
|  | **Stable MCI Aβ-** | <0.001 | 0.570 | 0.566 | - |
|  | **Stable MCI Aβ+** | <0.001 | 0.065 | 0.270 | 0.007 |

**Supplemental Table 2. Pairwise comparisons between groups**

Cohort 1: values depicted are p-values and p<0.05 signifies a significant difference. Cohort 2: depicted p-values are family-wise error corrected and p<0.05 signifies a significant difference. Differences in continuous variables were assessed using independent samples T-tests and differences in categorical variables with Fisher’s exact tests. Red shades cells indicate that the group on the x-axis was higher than the one on the y-axis and blue shading means the opposite.

|  | Janssen +  age, sex and APOE | Lilly +  age, sex and APOE |  |  |
| --- | --- | --- | --- | --- |
|  | **AUC** | **AUC** | **z-difference** | **P** |
| Aβ+ | 0.91(0.87-0.96) | 0.90(0.85-0.96) | 0.42 | 0.67 |
| AD-dementia | 0.92(0.87-0.96) | 0.93(0.88-0.97) | -0.49 | 0.62 |

**Supplemental Table 3. Predictive effects of plasma p-tau217 when also considering age, sex and APOEϵ4 carriership**

Effects were obtained by assessing the AUC of binary logistic regression models using ROC analyses.

|  | Plasma p-tau217+_Janssen_ |  |  |  |  | Plasma p-tau217_Lilly_ |  |  |  |  |
| --- | --- | --- | --- | --- | --- | --- | --- | --- | --- | --- |
|  | **Optimal cut point** | **AUC** | **Accuracy** | **Sensitivity** | **Specificity** | **Optimal cut point** | **AUC** | **Accuracy** | **Sensitivity** | **Specificity** |
| Aβ+ | 0.07  (0.05-0.08) | 0.85  (0.79-0.91) | 0.80  (0.75-0.86) | 0.75  (0.64-0.85) | 0.85  (0.75-0.95) | 0.26  (0.20-0.31) | 0.88  (0.81-0.91) | 0.83  (0.78-0.88) | 0.81  (0.69-0.92) | 0.85  (0.72-0.96) |
| AD-dementia | 0.08  (0.05-0.10) | 0.88  (0.83-0.91) | 0.82  (0.75-0.89) | 0.84  (0.71-0.98) | 0.81  (0.66-0.93) | 0.31  (0.25-0.39) | 0.89  (0.85-0.93) | 0.84  (0.78-0.9) | 0.85  (0.72-0.95) | 0.84  (0.72-0.94) |

**Supplemental Table 4. Sensitivity and specificity**

AUC, accuracy and sensitivity and specificity values with 95%CI were determined using the Youden index with bootstrapping (100 repeats).

**Supplemental Figure 1. Associations with cognition**

The top panel shows Spearman correlation analyses between baseline plasma p-tau217 of both assays and baseline MMSE. The bottom panel displays the associations between baseline plasma p-tau217 from both assays and annual change over time in MMSE, adjusted for age and sex. The z-statistics indicates results from a Fisher’s exact test assessing the difference between the correlation coefficient

**Supplemental Figure 2. Outliers removed for sensitivity analyses**

Outlier was determined by mean+/-3SD within diagnostic groups and denoted with an X

**Supplementary Text 1. Analytic performance of plasma p-tau217+_Janssen_ and plasma p-tau217_Lilly_ in cohort 2**

*Plasma p-tau217+_Janssen_*

The average lower limit of detection (LLOD) was 0.013 pg/ml. The analysis of both replicates failed for 8 samples due to instrument error and plasma p-ta217 value was below the LLOD for 1 of the remaining 277 samples. These 9 samples were excluded from the present study. For 74 samples (24%), only singlicate measurements were available because the analyses of one of the duplicated failed due to instrument error (n=72) or the p-tau217 value was below the LLOD (n=3). The average intra-assay coefficient of variation (CV) was 20.8% with CV<20% in 59% of cases. The average inter-assay CVs for 3 quality control samples analysed across all plates/runs was 10.1%.

*Plasma p-tau217_Lilly_*

The average LLOD for plasma p-tau217_Lilly_ was 0.15 pg/ml. Out of 303 samples included in the present study, 39 (13%) were below the LLOD. Of these 39, one case (with 1 sample) was associated with a CSF Aβ42/40 ratio <0.07 (i.e., CSF-AB+. The average intra-assay CV was 5.3% CV<20% in 96% of cases. The average inter-assay CVs for 3 quality control samples analysed across all plates/runs was 12.4%.
